# DNMT1 and p38γ are inversely expressed in reactive non‐metastatic lymph nodes burdened with colorectal adenocarcinoma

**DOI:** 10.1002/jha2.50

**Published:** 2020-06-29

**Authors:** Xu Hannah Zhang, Zhirong Yin, Aimin Zhang, Raju Pillai, Brian Armstrong, Steven T Rosen

**Affiliations:** ^1^ Department of Hematology City of Hope National Medical Center Duarte California USA; ^2^ Department of Pathology Solid Tumor Core City of Hope National Medical Center Duarte California USA; ^3^ Light microscopy core City of Hope National Medical Center, Duarte, California, City of Hope National Medical Center Beckman Research Institute Duarte California USA

**Keywords:** DNA‐methyltransferase 1 (DNMT1), p38γ (MAPK12: Mitogen‐activated protein kinase 12), Lymph nodes, germinal centers (GC), mantle zone of the follicle, The dark zone (centroblasts) of GC, The light zone (centrocytes) of GC, Chromatin Condensation Stage

## Abstract

Lymph nodes are important front‐line defense immune tissues, which also act against inflammatory diseases and cancer. Lymph nodes undergo extensive upheavals within newly formed germinal centers (GCs) when exposed to antigens, the molecular mechanisms of which remain elusive. Recently, p38γ was identified as an important target for multiple cancers, including cutaneous T‐cell lymphoma (CTCL). We previously observed that p38γ is overexpressed in CTCL versus normal cells, but it is not clear if p38γ is expressed in B or T lymphocytes of GCs of patients in response to a stress such as cancer. Therefore, in this study, we obtained non‐metastatic reactive lymph nodes adjacent to cancer lesions (colorectal adenocarcinoma), then performed multicolor immunohistochemical staining for p38γ and other relevant markers. We observed for the first time that p38γ was expressed in the light zone of activated B cells and T helper cells in GCs, whereas DNA‐methyltransferase 1 (DNMT1), a marker for GC B cells, was highly expressed in centrocytes and in the dark zone of GCs. This inverse relationship suggests a novel function for p38γ in T cells that cross‐talk to B cells in response to stress.

p38γ has emerged as an important target for multiple cancers with diverse pathways, including prostate,^1^ esophageal,^2^ breast,^3^ liver,^4^ and cutaneous T‐cell lymphoma (CTCL).^5^ It is overexpressed in malignant CD4^+^ T cells (ie, CTCL cells) but undetectable in normal healthy T cells. Whether p38γ expression is altered in the T cells of non‐metastatic lymph nodes (LNs) in response to stress is unknown, but understanding this will unravel questions about how stress is related to cancer, and ultimately achieve cancer prevention. Here, we performed multicolor immunohistochemical staining of p38γ (teal), DNA‐methyltransferase 1 (DNMT1; purple), and T‐ and B‐cell lineage markers CD4 (yellow) and CD20 (purple) using a Ventana Discovery Ultra system (Figure 1A‐D) on six swollen reactive regional LNs derived from a 47‐year‐old woman with a diagnosis of colorectal adenocarcinoma. The patient was treated with colectomy and swollen reactive regional LNs, obtained from pericolonic adipose tissues in which pathology confirmed no metastatic invasion, were chosen for the present study.

**FIGURE 1:**
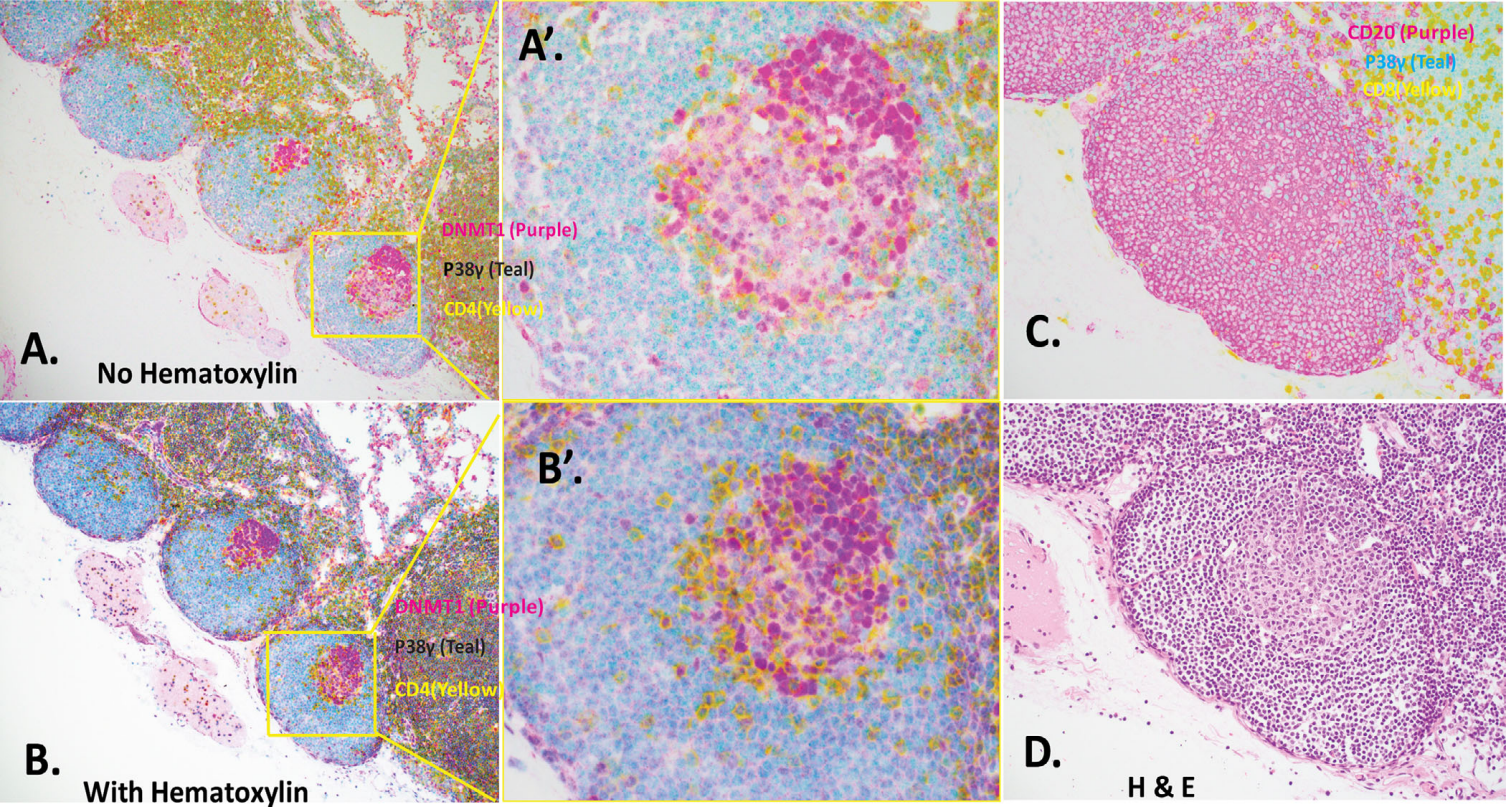
DNMT1 and p38γ expression in GCs of non‐metastatic lymph nodes. A,B, Sections showing three follicles within swollen regional lymph nodes removed from a patient with colorectal adenocarcinoma were stained for DNMT1 (purple), p38γ (teal), and CD4 (yellow) with no nuclear counterstain (A) or with hematoxylin nuclear counterstain (B). Magnification 10×, consecutive sections of the same lymph node paraffin block. A’,B’, Using the same images shown in A and B, the third follicle, indicated by yellow bracket, is visualized at 40× (A’) and (B’). C, Consecutive section stained with CD20 (purple), p38γ (teal), and CD8 (yellow). Magnification, 20×. D, Consecutive section stained with H&E for morphology and cell type verification

In colon cancer, the MAP kinase p38γ is overexpressed via the c‐Jun/MMP9 pathway [6, 7]. DNMT1, an essential marker in germinal center (GC) B cells for sustaining methylation status during DNA replication [8], transfers methyl groups to hemimethylated substrate CpG sites during DNA synthesis in S phase [9, 10, 11]. It is responsible for maintaining the genomic methylation pattern once de novo cell differentiation is established. Dnmt1 deletion in mice impairs Treg cells function and results in lethal autoimmunity [12]. To study the correlation between DNMT1 and p38γ, we first analyzed a public data set (Database GSE32018 / GPL6480 / A_24_P408083). We showed that the overall gene expression level of DNMT1 was higher in GC B cells in 22 diffuse large B‐cell lymphoma (DLBCL) patient samples in comparison to that of B cells in either 24 mantle cell lymphoma and 13 nodal marginal zone lymphoma patient samples (Figure S1), whereas p38γ expression was higher in mantle cell lymphoma than in DLBCL. This suggests that DNMT1 is negatively correlated with p38γ because the differential expression of the two genes is confined to different cell types within LNs (Figures 1 and 2).

**FIGURE 2:**
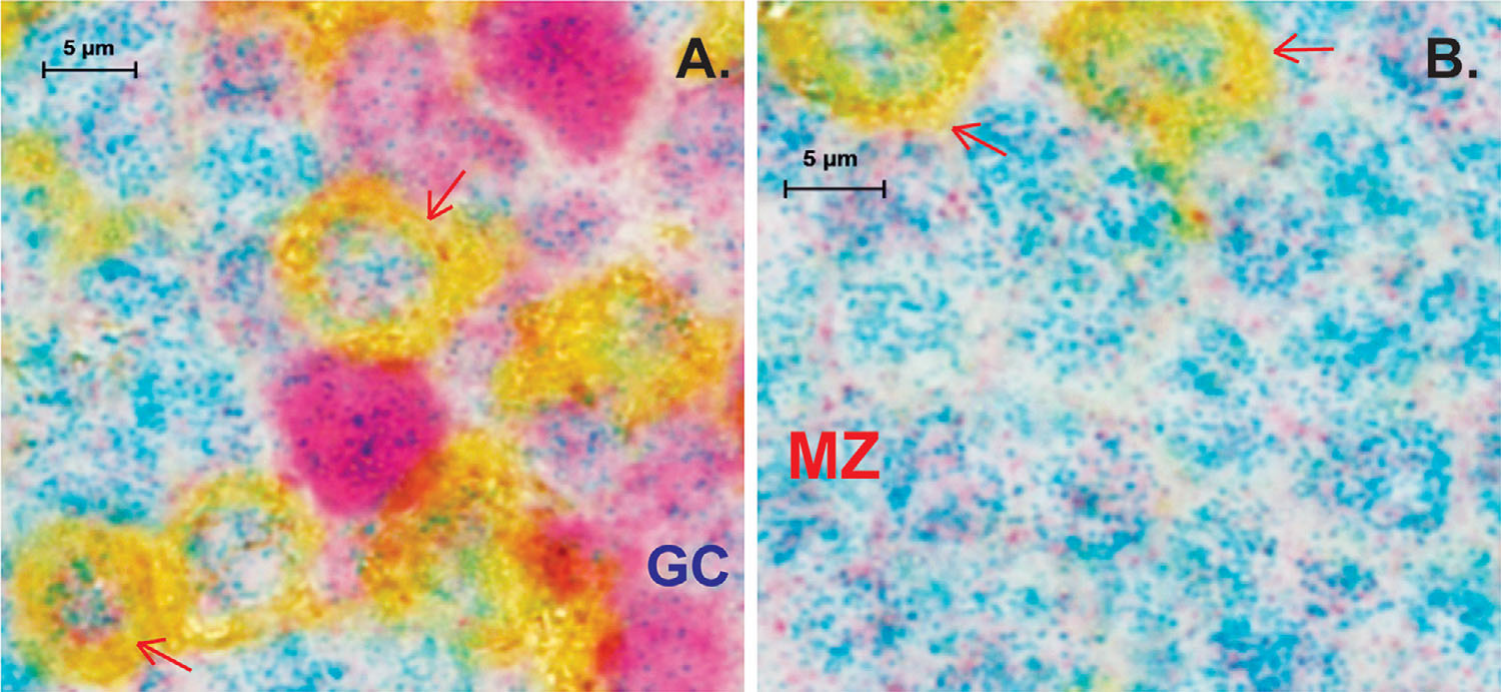
DNMT1 and p38γ expression in follicles of non‐metastatic lymph nodes. A, Elevated expression of p38γ exhibited in both germinal center B cells (Purple color) and CD4‐positive T cells (Yellow color) in the mantle zone with 40× magnification of Zeiss observer II microscopy analysis. p38γ expression (Teal color) is elevated in the nucleus of the T cells of mantle zone that adjacent to the GC and in that of GCB cells in reactive lymph nodes adjacent to solid tumors (red arrows, Green). GC B cells exhibits higher expression of DNMT1 comparing to that of mantle zone. B, The majority of cells in the mantle zone are resting B cells and in the nucleus of both T cells and B cells

We characterized the expression of p38γ in T cells within non‐malignant LNs during stress, such as cancer. Figures 1A (no nuclear counterstain) and 1B (with Hematoxylin nuclear counterstain) exhibit the same three LN follicles (10×) as consecutive slices of the same paraffin block, with the third follicle visualized at higher magnification (40× [Figure 1A’,B’]; middle images indicated by yellow bracket), stained for DNMT1, p38γ, and CD4. We found that DNMT1 was preferentially expressed in the dark zone (centroblasts) of GC B cells in a human LN, and to a lesser degree in the light zone (centrocytes; Figure 1A’,B’). p38γ was expressed in the mantle zone of the follicle, where the majority of cells were resting B cells (Figure 1A’,B’). The identity of mantle cells or GC B cells in the LN was further confirmed by CD20 (purple, Figure 1C) and H&E staining (Figure 1D) in consecutive slices. In contrast to p38γ, only a few DNMT1‐positive cells were observed in the mantle zone. In Figure 1A,A’, blue indicates co‐localization of DNMT1 (purple) and p38γ (teal) in GCs, while green shows co‐localization of p38γ (teal) and CD4 (yellow). Counter stain (Figure 1B’) strongly implicates that CD4‐positive (yellow) T cells are located at the border of the mantle zone and light zone.

In Figure 2, many CD4‐positive T cells were observed to show elevated expression of p38γ in the nucleus in the T‐cell zone of reactive LNs adjacent to solid tumors (Figure 2, red arrows). In addition, high expression of DNMT1 was observed in the B cells of a GC, suggesting a primary focus began to form when antigen was encountered in the follicle. p38γ mRNA expression was observed in naïve CD4^+^ T cells in healthy humans according to Monaco scaled RNA‐Seq signatures normalized by mRNA abundance [13] (Figure S2).

B cells in the mantle zone that surrounds a GC are in a “resting” stage; in contrast, GC B cells, where the affinity maturation and selection process occurs, are activated and proliferating. Affinity maturation selects B cells that have gone through somatic hypermutation, which alters the variable region of immunoglobin genes to select for those with high affinity for an antigen; the level of chromatin condensation at this stage is unknown. Figure 3 shows p38γ staining in the chromatin of mantle zone B cells, suggesting they are in a unique stage of hierarchical genome structure. Under normal physiological conditions, chromatin is compacted into varying levels of condensation according to each stage of the cell cycle, from relaxed chromatin fibers to its maximal mitotic condensation into chromosomes. Indeed, the observation that p38γ, which was expressed in the nuclei of B cells of the mantle zone and CD4‐positive cells in the T‐cell zone, where DNMT1 expression is low or non‐expressed, suggests a novel function of p38γ in T cells that cross‐talk to B cells in response to stress, such as post‐transcriptional or epigenetic gene regulation through suppression/blocking of expression of DNMT1.

**FIGURE 3:**
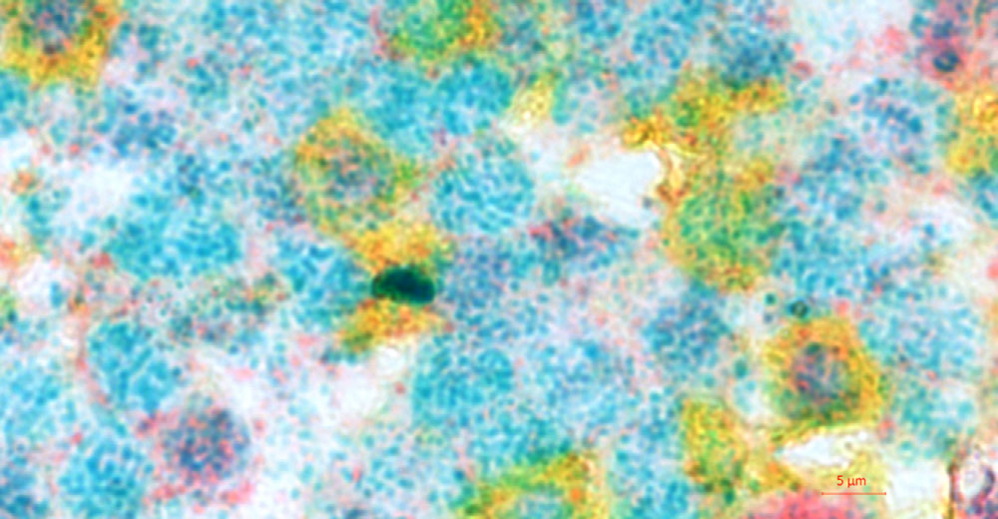
Chromatin condensed in the Mantle zone of LN that close to T cell zone (using 100× oil lens with Zeiss observer II microscopy).CD4: Yellow; p38γ: teal; DNMT1: purple; Blue: merged with teal and purple = p38γ^+^DNMT1; Green: indicates yellow merged with purple = CD4^+^ DNMT1; Red arrows indicates the three color merged: p38γ^+^DNMT1^+^CD4

For future perspective, this study is a probe for a big question: at what level of condensation can chromatin maintain a “resting” state in mantle zone cells, while remaining ready for generating new GCs through manipulation of methylation status in the mantle zone by the interplay of DNMT1 and p38γ?

## CONFLICT OF INTEREST

The authors declare no conflict of interest.

## Supporting information

Supporting Information.

Supporting Information.
