# Supplementary figures and images for: DNMT1 and p38γ are inversely expressed in reactive non‐metastatic lymph nodes burdened with colorectal adenocarcinoma
Source: EJHaem. 2020 Jun 29;1(1):300–3. doi: 10.1002/jha2.50 (PMC9176054; doi:10.1002/jha2.50)

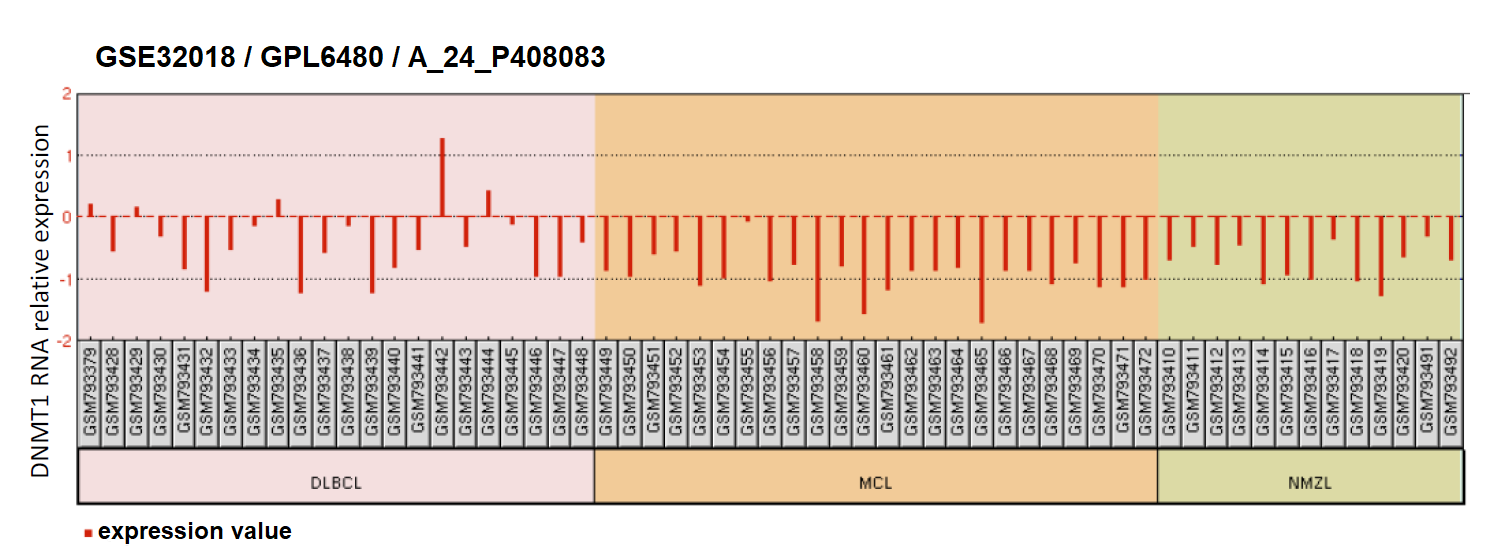

Supplement: Supplementary file 1 — Supporting Information. [file JHA2-1-300-s001.tif]

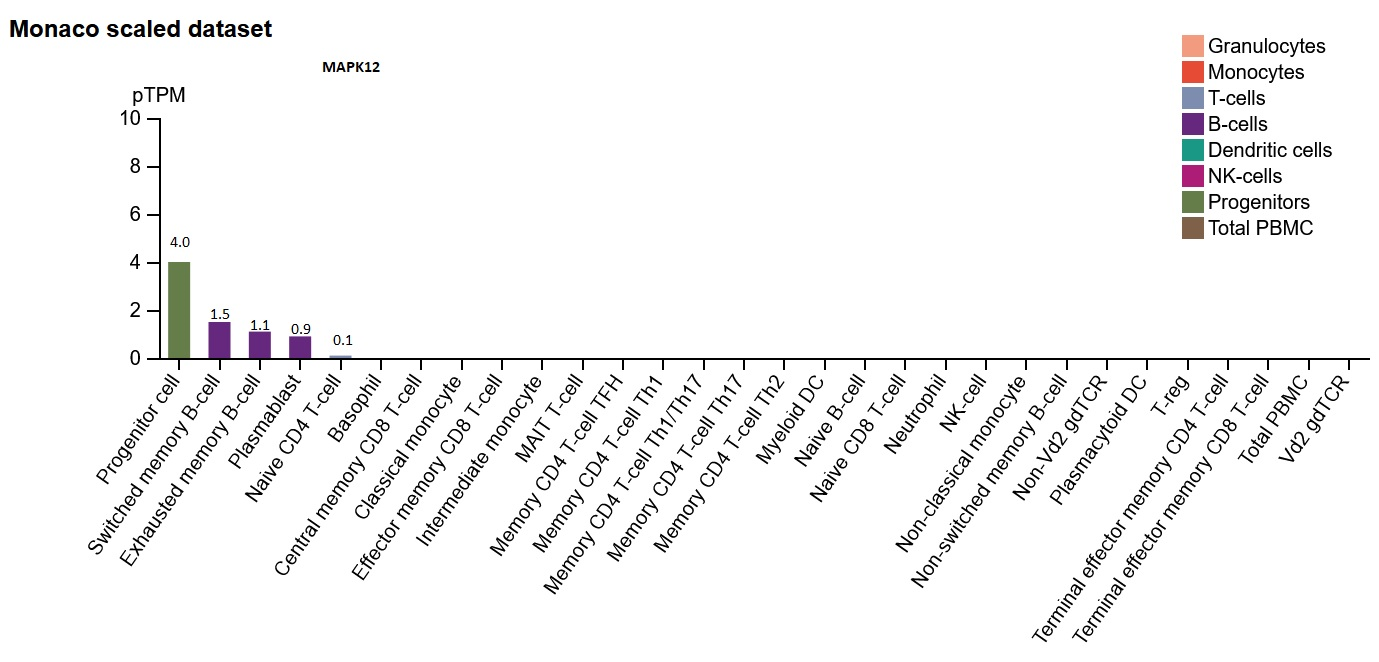

Supplement: Supplementary file 2 — Supporting Information. [file JHA2-1-300-s002.tif]
